# Solutions Trial: Solution Focused Brief Therapy (SFBT) in 10–17-year-olds presenting at police custody: a randomised controlled trial

**DOI:** 10.1186/s13063-024-07904-5

**Published:** 2024-03-02

**Authors:** Gwenllian Moody, Elinor Coulman, Emma Crocker-White, Kylie Gray, Richard P. Hastings, Andrea Longman, Fiona Lugg-Widger, Rebecca Playle, Jeremy Segrott, Paul Thompson, Julia Badger, Peter E. Langdon, Samantha Flynn

**Affiliations:** 1 Centre for Trials Research, Cardiff University, Neuadd Meirionnydd, Heath Park, Cardiff, CF14 4YS UK; 2 DECIPHer Centre, Cardiff University, Sparc Building, Maindy Road, Cardiff, CF24 4HQ UK; 3 Centre for Research in Intellectual and Developmental Disabilities (CIDD), University of Warwick, Warwick, CV4 7AL UK; 4 Brooklands Hospital, Coventry and Warwickshire Partnership NHS Trust, Birmingham, B37 5RY UK

**Keywords:** SFBT, Therapy, Intervention, Offending behaviours, Randomised controlled trial, Police custody, Children, Young people

## Abstract

**Background:**

Within England, children and young people (CYP) who come into police custody are referred to Liaison and Diversion (L&D) teams. L&D teams have responsibility for liaising with healthcare and other support services while working to divert CYP away from the criminal justice system but have traditionally not provided targeted psychological interventions to CYP. Considering evidence that Solution Focused Brief Therapy (SFBT) leads to a reduction in internalising and externalising behaviour problems in CYP, the aim of this randomised controlled trial (RCT) was to determine whether there is a difference between services as usual (SAU) plus SFBT offered by trained therapists working within a L&D team, and SAU alone, in reducing offending behaviours in 10–17-year-olds presenting at police custody.

**Methods:**

Design: two-arm individually RCT with internal pilot and process evaluation.

Participants: *N* = approximately 448 CYP aged 10–17 years presenting at one of three police custody suites in the area served by Lancashire and South Cumbria NHS Foundation Trust (LSCFT) who are referred to the L&D team. Participants will be recruited and allocated to intervention:control on a 1:1 basis. Interviews will be performed with 30–40 CYP in the intervention arm, 15 CYP in the control arm, up to 20 parents/guardians across both arms, up to 15 practitioners, and up to 10 site staff responsible for screening CYP for the trial.

Intervention and control: Those allocated to the intervention will be offered SAU plus SFBT, and control participants will receive SAU only.

Primary outcome: CYP frequency of offending behaviours assessed through the Self-Report Delinquency Measure (SRDM) at 12 months post-randomisation.

Secondary outcomes: criminal offence data (national police database); emotional and behavioural difficulties (self-report and parent/guardian reported); gang affiliation (self-report).

Process evaluation: evaluation of acceptability and experiences of the CYP, parents/guardians, site staff and practitioners; fidelity of SFBT delivery.

**Discussion:**

This two-arm individually RCT will evaluate the effectiveness of SFBT in reducing offending behaviours in CYP presenting at police custody suites within the area served by LSCFT. Our process evaluation will assess the fidelity of delivery of SFBT, the factors affecting implementation, the acceptability of SFBT in CYP aged 10–17 years and recruitment and reach. We will also examine systems and structures for future delivery, therefore assessing overall scalability.

**Trial registration:**

ClinicalTrials.gov ISRCTN14195235. Registered on June 16, 2023.

## Administrative information

**Table:** 

Title {1}	Solutions Trial: Solution Focused Brief Therapy (SFBT) in 10–17-year-olds presenting at police custody: a randomised controlled trial
Trial registration {2a and 2b}.	ISRCTN14195235 https://www.isrctn.com/ISRCTN14195235
Protocol version {3}	1.5 23.03.2023
Funding {4}	The trial is funded by the Youth Endowment Fund (YEF). Sites will meet the costs of programme delivery through funding from YEF.
Author details {5a}	1 Centre for Trials Research, Cardiff University, Neuadd Meirionnydd, Heath Park, Cardiff, UK, CF14 4YS. 2 DECIPHer, Cardiff University, Sparc, Maindy Road, Cardiff, CF24 4HQ. 3 Centre for Educational Development, Appraisal and Research, University of Warwick, UK, CV4 7AL. 4 Brooklands Hospital, Coventry and Warwickshire Partnership NHS Trust, Birmingham, B37 5RY.
Name and contact information for the trial sponsor {5b}	Andrew Pennington, Associate director R&D, Lancashire and South Cumbria NHS Foundation Trust E-mail: Andrew.pennington@lscft.nhs.uk
Role of sponsor {5c}	The sponsor has had no role in the design, nor the analysis and interpretation of data, nor the writing of the report and the final decision to submit the report for publication. The sponsor has assisted with the identification of potential participants, consent and collection of trial data and the delivery of the intervention as staff employed to undertake these tasks were employees of the sponsor.

## Introduction

### Background and rationale {6a}

When children are arrested in England many are referred to a Liaison and Diversion (L&D) team. This trial will take place within L&D teams in the area served by Lancashire and South Cumbria NHS Foundation Trust (LSCFT) where the L&D service is provided across 12 custody suites within this region. CYP who are assessed by the L&D team are not always the same children who are supported by statutory services such as children’s social care or child youth justice. The current trial provides an opportunity to test the effectiveness of Solution Focused Brief Therapy (SFBT) as a psychological intervention, aimed to divert CYP from serious youth violence and safeguard them from criminal exploitation in the community. The focus is on early intervention, often with children who are not already supported by statutory services. L&D teams have not traditionally offered any intervention as they are an assessment and signposting service with the goal of diverting CYP away from criminal justice services. There is no research demonstrating that SFBT, delivered by L&D teams, is effective in reducing future offending behaviours.

Iterations of the NHS Long Term Plan for England have had focus upon CYP, including those with mental health problems, and those who encounter criminal justice. One of the important aims of the NHS Long Term Plan was to further develop services to help CYP access treatment faster. This included expanding services to deliver them when and where CYP need them, which could include schools and colleges, as well as when they encounter the police. The objectives set for the NHS for 2020/2021 included an expansion of L&D services such that 100% of those who need this service receive this service [1]. As part of this, a significant expansion of high-quality mental health care for CYP was planned, meaning that an additional 70,000 CYP should have been able to access psychological therapies when and where needed by 2021 [1]. Under the long-term plan, services for CYP were set to expand within community-based settings with an increased focus upon timely and appropriate crisis support and intervention. The current trial fits with this policy landscape and overall goals as set by NHS England. At the same time, the current project fits with the vision set by the funder of this trial, the Youth Endowment Fund (YEF), which is to prevent CYP from becoming involved in violence [2].

### Rationale for current trial/justification of treatment options

SFBT helps people to change by focusing on building solutions rather than getting stuck thinking about problems. Its effectiveness across a variety of contexts and presenting problems has been supported by a number of systematic reviews and meta-analyses “e.g. [3, 4]”. SBFT is widely regarded as a flexible and inclusive approach which has been attributed to its emphasis on simplicity and use of brief interventions [5]. A recent systematic review describes SFBT as a culturally sensitive approach that has been applied worldwide and found to be effective across a range of cultures [6]. SFBT has also been found to be effective for males and females in improving internalising problems such as low self-esteem [7] and depression [8] as well as externalising child behaviour problems such as aggression [9, 10], truancy [11] and substance use [8, 12]. SFBT has also been found to be effective for individuals with intellectual disabilities [5] and those from all socioeconomic groups, including economically disadvantaged backgrounds [13]. Evidence suggests that SFBT may be particularly effective when presenting problems are less severe [3] which may also reflect the significance of its implementation as an early intervention programme for young people.

More broadly, psychological therapies within Europe that have a behavioural or cognitive orientation have been shown to be more effective at reducing recidivism amongst CYP than interventions focused upon increasing supervision or deterrents [14]. However, there is also evidence to suggest that community-based psychosocial interventions, and specifically, functional family therapy, group-based cognitive behavioural therapy, mentorship, multidimensional family therapy, and multi-systematic therapy are no more effective at reducing re-offending amongst adolescents than SAU [15, 16]; this finding may be related to the nature of SAU within some countries with state-funded health and social care, and indicates that descriptions of SAU should be included within trials that aim to test interventions designed to reduce re-offending amongst CYP.

A number of policies and guidelines have proposed the expansion of the existing L&D service, to include psychological therapies as appropriate for CYP at risk of engaging in violence and crime in the future [17]. Research has shown that SFBT can be an efficient therapy known for its, ‘briefness’ and cost efficiency [18], as well as its effectiveness in sustaining long-term outcomes which have been found to be comparable with other established therapeutic approaches such as cognitive behavioural therapy (CBT) [19, 20]. Considering the research outlined above it may be suggested that SFBT might be an effective and efficient approach to reduce the risk of CYP engaging in violence and crime in the future if provided shortly after arrest. This would be in addition to the current assessment and signposting work undertaken by L&D teams and research should explore the feasibility and effectiveness of SFBT implementation within this context.

### Objectives {7}

The primary objective of this trial is to determine whether there is a difference between SAU plus SFBT and SAU alone in reducing offending behaviours in 10–17-year-olds presenting at a police custody suite.

The secondary objectives are to:

- Complete an internal pilot in the first seven months of the intervention starting to examine whether moving to a definitive trial is warranted and feasible.
- Generate evidence to consider whether there is a difference between SAU plus SFBT and SAU alone on scores for Strengths and Difficulties Questionnaire (SDQ) [21] — externalising, internalising and prosocial behaviours at 12-month follow-up.
- Examine whether there is a difference between SAU plus SFBT and SAU alone on changes in offending behaviours, specifically the change in the numbers of arrests, cautions, reprimands, warnings, and convictions between baseline and 12-month follow-up.
- Examine whether there is a difference between SAU plus SFBT and SAU alone on the Gang Affiliation Measure (T-GARM) [22, 23] at the 12-month follow-up.
- Carry out exploratory sub-group analyses of the primary outcomes by intellectual disability status [24], and callous-unemotional traits [25, 26].
- Monitor and report any adverse events related to SFBT.
- Complete a process evaluation using key indicators drawn from the logic model, including an evaluation of intervention acceptability and the experiences of CYP, parents/guardians, and other key stakeholders (e.g. practitioners, delivery team), fidelity of delivery of SFBT, intervention reach and defining SAU.
- Explore the availability of routine data sources.
- Explore how any reduction in offending behaviour relates to critical moments of school exclusion.

### Trial design {8}

The trial is a two-arm individually RCT of SAU plus SFBT versus SAU alone, involving CYP (age 10-17 years old) who have presented at one of three police custody suites in the region served by LSCFT. The trial involves an internal pilot to be completed at month 12 of the trial, the set-up phase will last five months, and the pilot phase will last seven months (see section 12 for more details). Approximately 448 CYP participants will be recruited to the full trial. Participants will be randomised on a 1:1 basis to either intervention or control arm using permuted block randomisation, stratifying by Verbal Comprehension Index (VCI) [24] and custody suite.

## Methods: participants, interventions and outcomes

### Study setting {9}

The trial will take place within community-based settings. This trial will be carried out at one participating site within the UK: three custody suites in the geographical region served by LSCFT.

### Eligibility criteria {10}

Participants are eligible for the trial if they meet all of the following inclusion criteria and none of the exclusion criteria apply.

### Inclusion criteria

- 10–17 years of age
- Referred to the L&D Team by the police.

### Exclusion criteria

- A clinician has judged that the CYP is presenting with a mental illness of a nature or degree warranting immediate intervention from specialist services, including assessment for detention under the Mental Health Act.
- The CYP is to be remanded into custody.
- A CYP aged 16 years or older judged to lack the mental capacity to decide about participating in this trial by staff responsible for gaining informed consent.
- The CYP is living outside the area served by LSCFT.
- The CYP is unable to converse in English.
- Parents/guardians are unable to converse in English (at least one must be able to converse in English to complete parent/guardian measures).
- Parents/guardians judged to lack the mental capacity to make a decision about themselves or their child (if under 16 years of age) taking part in this trial by staff responsible for gaining informed consent.

### Who will take informed consent? {26a}

Informed consent will be taken by a Research Assistant based at site or with the evaluation team. The CYP and parent/guardian will have been sent Participant Information Sheets (PIS) and a copy of the Consent/Agreement Forms (CF) in sufficient time prior to meeting the Research Assistant. There will be two age-appropriate versions of the PIS for CYP, one for 10- to 13-year-olds, and one for 14-year-olds and older. Supplementary trial information will be provided in alternative formats (e.g. video, leaflet, poster, brief one-page PIS, participant journey document). If happy to take part, informed consent will be obtained from CYP 16 years of age and older; agreement will be obtained from CYP under 16 years of age once parental/guardian consent has been given. Verbal consent will be obtained (either via telephone, videoconferencing or face-to-face meeting). Research Assistants will complete an online consent form on behalf of the participant using REDCap e-consent while speaking with them, evidence of this will be a PDF screenshot sent to the participant. If a participant turns 16 during the course of the trial, they will be re-consented, before the next data collection/follow-up stage.

A contacts form will be completed for participants including multiple methods of contact (address, telephone, email address) to minimise loss at follow-up. Preferences for follow-up data collection (face-to-face, telephone, online, videoconferencing, or postal) will be obtained, but participants are free to change their minds about their preferred method and will be asked for their preferences at each data collection point.

Informed consent will be taken in the same way for qualitative data collection.

### Additional consent provisions for collection and use of participant data and biological specimens {26b}

Consent will be sought for data (including personal data and special categories including criminal offence data) to be archived at the end of the trial via the Office for National Statistics (ONS) Secure Research Service (SRS). Seeking this consent is a condition of taking part in the trial and a requirement of the funder. Data sharing plans will be explicitly included in the PIS.

## Interventions

### Explanation for the choice of comparators {6b}

The control group will receive SAU as currently delivered by L&D teams. This was chosen as it is current clinical practice governed by national guidance [27]. This includes the identification, screening, and assessment of vulnerable individuals leading to referral to mainstream health and social care services where they may be offered a variety of health and social care interventions depending upon their level of need and incorporating any necessary safeguarding action. SAU has been described elsewhere which includes offering advice, referral to health, psychiatric assessment, referral for psychological therapy, or substance misuse work amongst other interventions; it has been reported that long wait times to access onward services had a detrimental impact upon willingness to engage with these services [28].

### Intervention description {11a}

#### Solution Focused Brief Therapy (SFBT)

SFBT is a six-session manualised intervention, delivered face-to-face bi-weekly over 12 weeks, on a one-to-one basis, that helps people to change by focusing on building solutions rather than getting stuck thinking about problems. Through a programme of SFBT, it is hoped that CYP can be diverted away from the criminal justice system, reducing their risk of serious youth violence. The six sessions are detailed below.

##### Session 1: negotiating the contract

Introductions are made, with a focus on establishing a therapeutic relationship. The CYP is engaged in problem free talk, giving an opportunity to discuss positive aspects of the CYP’s life, rather than the problems that led to the referral to L&D. The CYP’s hopes for the session are established, boundaries are defined, and a confidentiality agreement is agreed upon.

##### Session 2: A preferred future

The session begins with problem free talk, including the CYP’s highlights since the last session. This session focusses on the miracle question, which asks the CYP to consider an alternate reality where things are different, better, and problems are resolved, and the CYP’s preferred future including exploring what aspects of this are already happening (e.g. identifying small positives from their everyday lives to sow the seed of hope for an alternative, and positive, future).

##### Sessions 3–5: using scales

Each session begins with problem free talk and reflection on the time between sessions, including any highlights. The preferred future from session two will be discussed and they will identify aspects of the preferred future that have been present since the last session. The therapist will then introduce the use of scales that will form the basis of sessions 3–5 (the CYP will rate themselves 0–10, with 10 being the most preferred outcome and 0 being the least preferred). The focus of the discussions will be the CYP’s position from a positive perspective. Any topic can be included.

##### Session 6: ending session

The focus of the final session is to end the therapeutic relationship safely. The therapist will ask about what is better for the CYP now, compared to session one. They will reflect on CYP’s improvements, using the scales as evidence. The therapist will work with the CYP to identify short-term goals post-intervention, as well as who their support team is to be their continued support.

The intervention will be delivered from months 6 to 19 of the trial. The therapists have been recruited from the existing L&D workforce within the area served by LSCFT. Practitioners are from a health and social care skill mix and are in band 5 or 6 clinical roles as per the Agenda for Change pay scales within the National Health Service. All three practitioners recruited to support the trial already have experience in supporting children through custody. For the trial, they have then undertaken 36 hours of SFBT training, facilitated by the same training provider at the same time.

### Criteria for discontinuing or modifying allocated interventions {11b}

The intervention can be discontinued at participants’ request, or following the occurrence of a related serious adverse event. The intervention can be modified or adapted while still working within the structure of the manualised guidance at the therapists’ judgement. There is space to record modifications in the fidelity checklist if therapists wish to do so.

### Strategies to improve adherence to interventions {11c}

While the SFBT intervention is to be delivered consistently as per the manual, there will be a degree of flexibility in terms of frequency, duration and venue to optimise engagement and adherence to intervention for young people, described below. This intervention is required to be delivered over a 12-week period with expectations of engagement of between no less than seven days or more than 14 days between sessions. Each of the six intervention sessions can last between 15 min and up to one hour. This is expected to be negotiated with each young person prior to the start of each session. The therapy is to be offered on a one-to-one basis with consideration for times where it may be appropriate for the young person to be accompanied by a family member or main carer. Adaptations to support adherence to the intervention will involve offering the CYP the opportunity to meet at a range of community settings for the intervention. These adaptations will be recorded, and a record kept of anyone who attends with the CYP.

### Process evaluation

The process evaluation will aid interpretation of trial outcomes by examining four key aspects of intervention implementation: (1) recruitment and reach; (2) intervention delivery, including adherence and fidelity; (3) factors influencing intervention implementation, (4) intervention mechanisms. MRC guidance will be used as a framework for the process evaluation.

### Recruitment and reach

Demographic and baseline data will be used to describe the CYP. Screening logs and withdrawal data will be used to record how many CYP were approached, the proportion that were recruited, and how many were retained at all stages and reasons for attrition (if given). Ethnicity age, sex, and VCI will be entered into our interview sampling framework. A framework will be used, including session attendance and therapist.

### Implementation fidelity/adherence and dosage

SFBT attendance/engagement data will be recorded in logs by practitioners, including the start date of CYP engagement with the intervention, and the number of sessions offered and completed to record dosage. CYP engagement in sessions will also be recorded. The number of sessions delivered will be recorded by practitioners in Session Summary forms and any implementation challenges recorded. Advice was taken from the Project Advisory Group (PAG, made up of CYP in the area served by LSCFT) to ascertain whether audio/videorecording of sessions or a researcher/practitioner observing sessions would be acceptable to CYP as a method to capture fidelity data. The advice was that neither method was acceptable. Consequently, a practitioner-completed fidelity checklist was developed collaboratively with therapists. The fidelity checklist is comprised of items representing the core components of the BRIEF SFBT model. These include building rapport and setting expectations for therapy, adopting a ‘not knowing’ stance, incorporating client hopes for the future, and the use of the miracle question, amongst other components, including a record of any adaptations made to therapy. Quantitative data on adherence and fidelity will be used for analysis of key trial outcomes, to investigate relationships between intervention outcomes and intervention receipt, adherence, and fidelity.

### Factors influencing intervention implementation and reach

Interviews with 30–40 CYP (up to 15 per custody suite) in the intervention group will be completed to ascertain their experiences of taking part in the trial (e.g. randomisation, questionnaire completion), receiving SFBT, and factors impacting adherence to intervention. Interviews with up to 15 CYP in the control group will also be completed to ascertain their experiences of taking part in the trial and this will be balanced across custody suites where possible. All interviews will explore retention to the trial, and factors affecting this. CYP will be sampled to ensure a spread of CYP age and referral custody suite. Semi-structured telephone/online interviews with up to 20 parents/guardians across both arms of the trial from across the three custody suites will be completed to gather in-depth data about their experiences of the trial, attitudes/perceptions of SFBT, and factors impacting adherence to intervention (if their CYP was in the intervention arm). Interviews with up to 15 practitioners will explore their experience of delivering SFBT and the potential systems and structures which would be needed for future implementation of SFBT. Interviews with CYP, parents/guardians and practitioners will also explore the provision of existing services (SAU) and how SFBT is distinct from this provision. Interviews with up to 10 site staff who are not practitioners but who will screen CYP who might want to take part in the trial will also be conducted to explore their experiences and views of the trial and the intervention.

### Intervention mechanisms

Interviews with up to 15 practitioners will also explore factors impacting adherence and fidelity, which will help us to understand the mechanisms that might contribute to/explain the outcomes of the trial. Qualitative interviews with CYP and parents/guardians will explore the perceived benefits and mechanisms of the interventions. Qualitative interviews with practitioners will explore unintended effects and key components of SFBT. These data will enable us to explore the extent to which key intervention mechanisms appear to be working as intended, variation across context (e.g. by practitioner, custody suite, family context), and any unintended mechanisms or barriers to participation. Together with quantitative data on hypothesised short-, medium-, and long-term impacts, these data will be used to refine the intervention’s logic model and to examine ways in which SFBT adds to and/or strengthens potential impacts of SAU.

### Interview process

Interview topic guides for all participant groups will be informed by the previous literature, quantitative outcomes (where relevant), fidelity/adherence data, and iteratively throughout the interviewing period whereby interview questions may evolve to address questions that arose in previous interviews. Interview topic guides will be reviewed by members of the Trial Management Group, and members of the Project Advisory Group, with feedback on the guides being used to strengthen their validity.

Research Assistants will complete the qualitative interviews under the supervision of the co-Chief Investigator (SF), who in turn will be mentored by an expert in process evaluation methods (JS). Research Assistants will receive training prior to completing any interviews, and their initial interviews will be reviewed by SF, with feedback being given ahead of further interviews taking place to support high-quality interviews being undertaken consistently.

Interviews will be transcribed verbatim by experienced members of the trial administrative staff, and transcripts will be checked by Research Assistants against the original recording to ensure that the transcripts are reliable records of the audio recordings. Once this process is complete, audio recordings will be deleted.

Research Assistants will receive training in Framework Thematic Analysis prior to undertaking the analysis under the supervision of SF, who will continue to be mentored by JS. Reliability checks, whereby two members of the research team will independently code transcripts, will be undertaken on selected transcripts during the early stages of the analysis process before the remainder of the transcripts are analysed. A selection (up to 20% if needed) of the remaining transcripts will be coded by two members of the research team to ensure continued consistency throughout the analysis process. Should any disagreements in coding arise throughout this process, then the two researchers will attempt to resolve them through discussion, involving a third, independent member of the research team if disagreements cannot be resolved. Research Assistants will regularly discuss the analysis with SF, who will maintain involvement at each stage to ensure that the process is being completed with a high degree of reliability. An emergent framework will be developed by Research Assistants and SF, which will be agreed with the wider Trial Team. Before being finalised, the emergent framework will be presented to members of the Trial Management Group, Trial Steering Committee, and Project Advisory Group who will be encouraged to provide feedback on the findings to encourage further considerations about the analysis for the research team. This will provide a further opportunity for the research team to strengthen the validity of the findings.

### Relevant concomitant care permitted or prohibited during the trial {11d}

SAU will be delivered to all participants enrolled in the trial. There are no prohibitions on the delivery of additional interventions.

### Provisions for post-trial care {30}

There are no provisions for ancillary or post-trial care other than SAU. The NHS indemnity scheme applies and provides cover against harm arising from clinical negligence in the conduct of research. NHS indemnity arrangements do not extend to non-negligent harm.

### Outcomes {12}

#### Primary outcome measure

The proposed primary outcome measure for this trial is the Self-Report Delinquency Measure (SRDM) at 12 months post-randomisation [29], which is a short measure comprising 15 items pertaining to antisocial behaviours (e.g. burglary, violence). This measure is relevant as it provides an index of the frequency of offending behaviours over time, including those that may not be known to the police. Comparisons between the intervention and control groups will be made at the primary endpoint, adjusted for baseline scores. It requires CYP to respond yes or no with reference to a time period (6 months). They then report the estimated frequency of the behaviour, and whether they have ever been caught. There is evidence that asking respondents to indicate whether they have engaged in these behaviours is accurate [30, 31]. We will compare SRDM mean scores between arms at 12 months post-randomisation, adjusting for baseline SRDM score, VCI, and custody suite.

#### Secondary outcome measures

Secondary outcome measures include:

- Criminal offence data: with consent from parents/guardians and CYP we will work with referrers and the police to gain access to arrest, caution, reprimands, warnings, and conviction data for participants (data held in the Police National Computer [PNC]). Crime data will be collected over the 6-month period prior to the commencement of treatment and at the 12-month follow-up. Comparisons between the two arms will be made at the primary endpoint adjusted for baseline data. These data are relevant because they provide an index of known criminal offending behaviour.
- Emotional and behavioural difficulties: the parent/guardian and self-report versions of the SDQ [21] will be used to assess CYP internalising, externalising, and prosocial behaviours. The SDQ is a robust and well-validated measure of behavioural and emotional problems measured over the preceding 6 months. This measure is relevant because it provides an index of emotional and behavioural difficulties that are thought to change as a consequence of treatment. Comparisons between the two arms will be made at the primary endpoint, adjusted for baseline scores.
- Gang Affiliation: T-GARM [22, 23] is a 26-item measure of gang affiliation that was developed with teenagers. Again, the scores on this measure will be compared at the primary endpoint, adjusted for baseline scores.

#### Potential moderators

In addition to the primary and secondary outcomes, potential moderators of the intervention will be examined:

- Callous and unemotional traits: This will be measured, at baseline and 12-month follow-up, using the 24-item Inventory of Callous and Unemotional Traits — parent/guardian report and youth self-report versions [25] which are robust and well-validated instruments [26]. It is thought that CYP who present with increased callous and unemotional traits may have different outcomes than those who presented with decreased levels of callous and unemotional traits.
- Intellectual disabilities: CYP will be invited to complete two subtests of the Wechsler Abbreviated Scale of Intelligence-II (WASI-II) [24] to index their VCI at baseline only. This scale is to be administered by a researcher (face-to-face, telephone, videoconferencing). The two subsets to be included are vocabulary and similarities. We are asking all participants to complete this measure as it is a stratification factor for randomisation. Further, verbal reasoning skills are thought likely to relate to treatment outcomes as the intervention is delivered using spoken communication within a therapist-participant dyad. A closed question asking if the child has an intellectual disability (parents and children will be asked this) will also be included at baseline, this question is taken from the Millennium Cohort Study [32]. These measures are essential to randomisation.
- Parent/guardian reports on other therapies their CYP is receiving (including pharmacological) will also be collected for demographic reporting.

### Participant timeline {13}

Schedule of enrolment, interventions and assessments

**Table:** 

Procedures	Data collection timepoints				
	Screening	Baseline	Treatment phase	6-month follow-up	12-month follow-up
Screening logs	X				
Eligibility	X				
Informed consent/agreement	X				
Contacts form	X				
Demographics		X			
Randomisation		X			
Delivery of intervention			X		
Compliance			X		
Outcome measures:					
CYP current case management		X			
Self-Report Delinquency Measure (SRDM) self-report [29]		X		X	X
CYP wellbeing self-report: self-report version of the Strengths and Difficulties Questionnaire (SDQ) [21]		X		X	X
CYP wellbeing parent/guardian-report: parent-report version of the Strengths and Difficulties Questionnaire (SDQ) [21]		X		X	X
Gang Affiliation Measure (T-GARM) (Gang Affiliation: T-GARM [22, 23]		X			X
Self-report Callous and Unemotional Traits [25]		X			X
Parent/guardian-report Callous and Unemotional Traits [25]		X			X
LD: Wechsler Abbreviated Scale of Intelligence-II (WASI-II) [24] (Vocabulary and Similarities Subtests). Question about if child has an intellectual disability.		X			
Parent/guardian-report other therapies received (including pharmacological)		X			X
Criminal offence data: arrest, caution, reprimands, warnings and conviction data (referrers and the police)		X			X
Fidelity measures:					
Attendance/engagement logs			X		
Session summary forms			X		
Qualitative interviews (post 6-month follow-up):					
• CYP				X	X
• Parents/guardians				X	X
• Practitioners				X	X
• Site staff				X	X
Withdrawal forms		X	X	X	X

### Sample size {14}

Approximately 448 CYP participants will be recruited allowing for up to 20% attrition (*N*=359). Recruiting this number of CYP, and on the basis of detecting a minimal clinically important difference (MCID) 0.325 (mean difference of 4 points with SD=12.32), assuming a correlation between baseline and follow-up of 0.334 [33] and using a two-sided alpha of 0.05, the trial would then be 90% powered. Our assumptions about the minimally detected effect size (MDES) are informed by previous research by the developers of the SRDM measure [34]. They report mean and standard deviation in the development samples and based on expertise in our target population have made a conservative adjustment to use a smaller MDES to reflect some level of uncertainty. We have also included the pre-post correlation but again there is minimal reported information in the extant literature, so we have made a conservative estimate on that basis. Sample size calculations will not be revisited after the internal pilot. The sample size has been designed to address the primary analysis only. Approximately 448 parents/guardians will be recruited for the main study, and up to 15 practitioners and 10 site staff will be recruited for qualitative data collection.

### Recruitment {15}

There will be one pathway for recruiting participants within L&D services in the site. Practitioners in L&D services will identify potential CYP participants who come into three custody suites within the area served by LSCFT. Potential CYP and parents/guardians (for CYP under 16 years of age) will be provided with trial information (either physically or by post/email) including a PIS, copy of the CF and contact information for the site staff. The screening and eligibility log will be completed by site staff. The screening log will also contain details of how a participant would prefer to complete the questionnaire (mode of completion). If the CYP (and parent/guardian if appropriate) are interested in taking part, an appointment will be arranged with a Research Assistant (via telephone or videoconference) and the following will be carried out:

- ◦ The trial will be explained in detail, including the randomisation and consent process. Research Assistants will ensure that the participant has had sufficient time to consider the information in the information pack.
- ◦ Eligibility will be confirmed.
- ◦ Consent to participate will be obtained from either:
- ◦ CYP parent/guardian alongside agreement from CYP if CYP is under 16 years of age
- ◦CYP consent from young people 16 years of age or older (in these instances we would also ask for consent to participate from the parent/guardian to complete the parent/guardian questionnaires; however, CYP 16 or older can still take part regardless of whether their parent/guardian takes part).

The appointment with the Research Assistant can be made in two ways, the Research Assistant can contact the participant/parent/guardian to arrange the appointment (using contact details from the screening log), or the participant/parent/guardian can get in touch directly with the Research Assistant to request the appointment (via contact details on the PIS, leaflet, poster, or brief PIS or via the trial website). If the participant is eligible and has been recruited to take part in the trial, their consent and contact details will be securely transferred to the trial team. Where a young person under 16 attends custody suite without a parent/guardian, then the young person can be sent home with information about the trial to give to the parent/guardian and the L&D team will follow up with the parent/guardian via telephone to ask if they are happy for the child to take part.

Both CYP and parent/guardian will be offered shopping vouchers for taking part in this trial, contingent upon questionnaire completion at each time-point (CYP baseline=£10, parents/guardian baseline=£10). In addition, some participants may have difficulties with reading and writing. We will make materials available in supplementary formats and the materials will be written in easy-to-read, lay language. Furthermore, we have adapted the trial materials for CYP to ensure that they are age-appropriate. Participants will be given the choice of how to complete the follow-up questionnaires (with a Research Assistant face-to-face, over the telephone, or via videoconferencing, or directly in the secure bespoke online database).

## Assignment of interventions: allocation

### Sequence generation {16a}

The randomisation sequence was generated using the STATA command *ralloc* using random permuted blocks (sizes 2, 4, 6) stratified by VCI and custody suite.

### Concealment mechanism {16b}

The online system ensures allocation concealment for the researchers recruiting participants, this is done by restricting access to the randomisation forms in the database from the researchers collecting data. Participants are also asked during follow-up interviews not to disclose their allocation to the researcher.

### Implementation {16c}

The randomisation system will be embedded within the REDcap online trial database. After completion of the baseline measures, participant details needed for randomisation (non-identifiable) will be passed from the Research Assistant to the Trial Manager or other trial representative, who will complete the randomisation online and inform the intervention practitioners of a participants’ allocation via secure file transfer.

## Assignment of interventions: blinding

### Who will be blinded {17a}

Due to the nature of the trial, participants and practitioners will not be blind to the allocation arm. In addition, the Trial Manager, Data Manager, Senior Trial Manager, and researchers completing qualitative interviews will not be blind to allocation. All other researchers, including the Trial Statistician responsible for analysing the data and researchers carrying out data collection, will be blind to the allocation arm. If inadvertent unblinding occurs during contact with a participant, this will be recorded and reported to the Trial Manager.

### Procedure for unblinding if needed {17b}

We do not foresee any circumstance where unblinding will be necessary and do not have an associated procedure.

### Data collection and management

#### Plans for assessment and collection of outcomes {18a}

Participants will be screened at the site, online or via the telephone and eligibility will be assessed. Potential participant details will be passed from the trial site to the trial team. The trial team will contact the participant as per their preferred method, to take consent and complete the baseline data:

- Baseline demographic case report form (CRF) including:
- Date of birth (DOB) (month and year only)
- Sex/gender
- Who they live with any changes in living arrangements between baseline and follow-up, and if they are in the care of the Local Authority
- Whether they are in school
- Type of school
- School year
- If not in school, whether they are working, training or in an apprenticeship, in college or out of work
- Ethnicity
- If English is their first language
- GP contact details

See section 12 for details of the proposed outcome measures.

Baseline outcome measures including (WASI-II [24] are to be completed with researcher assistance [telephone, videoconferencing, or face-to-face]). The trial team will also collect contact details including name, address including postcode, telephone number and email address for the purpose of completing follow-up. These will be kept separate from trial data. The trial team will make use of text messages, email, post, and WhatsApp messages, to maintain contact with participants and remind them of upcoming appointments. Full DOB will also be collected as the trial team will send participants a birthday card during the course of the trial.

After completion of the baseline measures, participant details will be passed from the Research Assistant to the Trial Manager or other trial representative and the participant will be randomised.

Participants will be followed-up at 6 months post-randomisation and 12 months post-randomisation.

We will also work with the provider team to ascertain what routinely collected data are available that can be used to inform our evaluation further (e.g. specific risk assessment measures). It will be explored whether the Ministry of Justice (MoJ) data linked to the Department for Education (DfE), which is available via the Data First collaboration with Administrative Data Research UK (ADRUK), would be available for this trial. Consent will be obtained for future linkage to the DfE dataset which will then be submitted to the ONS for storage in the YEF Data Archive.

### Plans to promote participant retention and complete follow-up {18b}

Participants who do not complete the 12-month follow-up data collection will be considered lost to follow-up. The trial team will monitor retention throughout the trial. In order to minimise loss to follow-up, participants (both CYP and parent/guardian) will be offered shopping vouchers for taking part in this trial, contingent upon questionnaire completion at each time-point, which will be stepped for CYP to encourage completion at the follow-up timepoints (CYP and parent/guardian: baseline=£10, 6 months=£15, 12 months=£20). CYP and parents/guardians will also be offered £20 shopping vouchers for participating in an interview. CYP and their parents/guardians will be sent thank-you cards following each contact. In addition, some participants may have difficulties with reading and writing, we will make materials available in alternative formats (see earlier) and provide a choice of data completion methods (see earlier). The materials will be written in easy-to-read, lay language and reviewed by the PAG. Furthermore, we have adapted the trial materials for CYP to ensure that they are age-appropriate. Participants will be given the choice of how to complete the follow-up questionnaires (see earlier). Participants will be sent email or text message reminders that their next assessment is due, and a reminder if the assessment has not been completed in a certain number of days. A fixed number of reminders will be sent as not to burden participants with reminders.

### Data management {19}

Source data will be paper or online versions of the CRFs/questionnaires. Participants are given the option to complete the CRF/questionnaire directly in the secure bespoke online database. If CRFs/questionnaires are completed by the Research Assistant face-to-face, over the telephone, or via videoconferencing the Research Assistant will complete the questionnaire on a tablet directly onto a secure bespoke online database. The Research Assistant will also be able to complete a paper copy of the CRF in case of technical difficulties. If CRFs/questionnaires are posted to the participants, they will be returned in free-post envelopes to the University premises where the data can be inputted by trial team staff. CRFs/questionnaires will only contain a unique identifier (PID) per participant, initials and partial DOB (month and year only). No other identifiable information will be recorded on the CRFs/questionnaires.

The trial team at the University of Warwick will enter paper CRF/questionnaire data on to the secure bespoke online database if required. Access to the database will be via username and password and restricted to appropriately trained personnel only. The database will be housed on local servers managed by Cardiff University in accordance with all appropriate legislation.

Identifiable data will be encrypted and stored separately from non-identifiable data with restrictions placed on members of the trial team without an honorary NHS contract meaning they will not have access to identifiers.

Wherever possible, data will be validated at point of entry, thereby reducing the opportunity for missing or unexpected data. All changes made to the data will be recorded and visible via an audit log within the database.

The planning, development, testing and maintenance of the database will be performed in line with CTR Standard Operating Procedures (SOPs), as will the data management function. Copies of CRFs/questionnaires will be returned to the CTR/Trial Manager by courier or scanned and sent via secure file transfer. Qualitative interview recordings will be recorded on encrypted audio-recorders/video-recorders/videoconferencing and stored on password-protected computers at Warwick. Recordings will be transcribed at the University of Warwick and securely transferred to the trial team at Cardiff University by secure file transfer. All files will be encrypted. Identifiable information will be removed from transcripts, transcripts will be pseudonymised, and original recording will be deleted as soon as the transcripts have been completed and checked.

A data management plan has been developed to outline the details of how data will be collected, transferred stored and accessed by the team.

### Confidentiality {27}

The trial team will act to preserve participant confidentiality and will not disclose or reproduce any information by which participants could be identified, except where specific consent is obtained. Data will be stored in a secure manner and will be registered in accordance with the General Data Protection Regulation (GDPR) 2016. We must ensure that it is use of personally-identifiable information will be in the public interest and used in accordance with the GDPR. Data will be collected from data providers such as the police (on the PNC), and data will be shared with DfE and ONS.

Participants will always be identified using a unique Participant Identification (PID) number and additional identifiers. All other identifiable information will not be stored with collected data.

### Plans for collection, laboratory evaluation and storage of biological specimens for genetic or molecular analysis in this trial/future use {33}

N/A — no biological specimens will be collected.

## Statistical methods

### Statistical methods for primary and secondary outcomes {20a}

The primary analysis will include all randomised participants in the groups to which they were originally allocated, irrespective of treatment received, who provide outcome data (i.e., an intention to treat analysis set). Mean SDRM scores will be compared between arms at 12 months post-randomisation using linear regression, adjusting for baseline SRDM score, VCI, and custody suite. Effect sizes as Hedges’ g (adjusted mean difference [35]) will be reported and, in addition, all estimates will be reported with their associated 95% confidence intervals.

Secondary outcomes will be analysed following a similar framework. The parent/guardian and self-report versions of the SDQ will be analysed following the same model as our primary outcome. The distributions of these secondary outcomes will be assessed prior to conducting the analysis. If skew is significant and residual assumptions are not met, then a Poisson or Negative Binomial model will be specified. If range restriction is apparent (significant floor and ceiling effects in distribution plots), then a Tobit regression [36] will be used. The remaining secondary outcomes, the number of criminal offences, will be analysed similarly but use generalised linear mixed models (GLMM).

All analyses will be checked subject to satisfying the required assumptions.

If distributional assumptions are not satisfied, as appropriate, a generalised linear model with an alternate link function will be used.

We will conduct two sensitivity analyses:

- Exploring the impact of missing data on trial outcomes
- Exploring the impact of different levels of intervention receipt on outcomes. We will use either two-stage least squares instrumental variables regression or inverse probability of treatment weighting methods to examine the effect of the intervention in those who receive varying levels of it.

We will describe process evaluation measures and fit regression models whereby we explore their association with outcomes. As these will only be measured in those allocated to the intervention, these will be associational in nature.

A final Statistical Analysis Plan (SAP) will be produced prior to any analysis being undertaken and will provide details of handling missing data.

### Interim analyses {21b}

No interim analyses will be undertaken. Beyond the internal pilot, there will be no formal ‘stopping rules’ or ‘discontinuation criteria’ for individual participants, parts of the trial and the entire trial. Any concerns with participant well-being will cross reference this section with those from the Trial Steering Committee (TSC) as this group is likely to be involved with this decision-making process.

The continuation of the trial from the internal pilot to the main trial will be decided by the TSC and funder at month 12.

### Methods for additional analyses (e.g. subgroup analyses) {20b}

The extent to which there may be differential intervention effects by custody suite will be explored by extending our primary analysis model to include sub-groups by trial arm interaction terms. Similarly, potential moderators (intellectual disability status and callous-unemotional traits) will be explored by the inclusion of an interaction of moderator and treatment allocation variables into the primary analysis model. As a further secondary step in the analyses, we will also explore whether age and sex covariates influence outcomes (adjust estimates) by inclusion as covariates in the linear regression model.

The role of the therapist as a source of clustering in the intervention arm will also be considered. To account for any clustering in the intervention arm, the statistician will fit a heteroscedastic partially nested mixed-effects model structure.

The statistician will fit linear mixed models, accounting for repeated post-randomisation measures (6 and 12 months post-randomisation) within participants, adjusting for baseline measures, custody suite and practitioners to investigate the overall effect of the intervention on post-randomisation measures.

Exploratory mediation analyses may also be carried out to examine variables at 6 months that may mediate intervention effects between baseline and 12-month follow-up. Any such analyses will be specified once a final logic model is confirmed.

### Methods in analysis to handle protocol non-adherence and any statistical methods to handle missing data {20c}

The impact of missing data on trial outcomes will be explored by investigating likely missing data mechanisms and re-fitting the primary outcome within a multiple imputation framework (including exploring missing mechanisms via delta-based controlled multiple imputation). Any deviations from the original SAP will be submitted as substantial amendments where applicable and recorded in subsequent versions of the protocol and SAP.

### Plans to give access to the full protocol, participant-level data and statistical code {31c}

Requests for access to the full protocol document will be granted. Data will be archived at the end of the trial via the Office for National Statistics (ONS) Secure Research Service (SRS). Requests for statistical code will be considered.

## Oversight and monitoring

### Composition of the coordinating centre and trial steering committee {5d}

The Centre for Trials Research in Cardiff University is co-ordinating the trial.

Those responsible for the day-to-day running of the trial, inclusive of the Research Assistants, key members of the Centre for Trials Research in Cardiff University, other site staff, and Chief Investigators, meet fortnightly.

A TSC, consisting of an independent chair with relevant expertise, and at least two other independent members including a Lay Representative and Statistician, will meet at least annually and will oversee all aspects of the trial. Non-independent members will include the joint Chief Investigators (CIs). The Statistician, Trial Manager and other members of the trial management team may attend in an observer capacity at the request of the Chair.

The TSC will provide overall supervision for the trial, provide advice through its independent chair and will be held at least four points throughout the duration of the trial. The ultimate decision for the continuation of the trial lies with the TSC and funder. TSC members will be required to sign up to the remit and conditions as set out in the TSC Charter.

The Trial Management Group (TMG) will meet at least quarterly during the trial. TMG members will consist of all Co-investigators, collaborators and the trial team and will oversee all aspects of the trial. The role of the TMG will be to provide specialist advice, input to and comment on trial procedures and documents (PIS, protocol, etc.). They will also advise on the promotion and running of the trial and deal with any issues that arise. TMG members will be required to sign up to the remit and conditions as set out in the TMG Charter. 

The PAG will be responsible for providing advice on all trial aspects from the perspective of young people in similar circumstances, including study materials, social media campaigns, videos, and recruitment strategies. The LSCFT site will assist in finding appropriate members for this group. This group will meet on a regular basis and will assist the trial as needed. A lay representative will sit on the TSC.

### Composition of the data monitoring committee, its role and reporting structure {21a}

The TSC will be responsible for determining if a Data Monitoring Committee (DMC) is required for this trial. If a DMC is deemed necessary, DMC members will be required to sign up to the remit and conditions as set out in the DMC Charter. It was agreed with the funder that no DMC would be necessary due to the low-risk nature of the trial. The TSC will take DMC responsibilities if they deem a DMC to be unnecessary.

### Adverse event reporting and harms {22}

Expected adverse events will be assessed by the TSC and reported to the Research Ethics Committee (REC) for consideration, as required. All serious adverse events (SAEs) will be reported immediately (and within 24 hours of knowledge of the event) by the Principal Investigator (PI) at the participating site to the trial team unless the SAE is specified as not requiring immediate reporting.

#### Definitions

This trial will collect Good Clinical Practice (GCP) SAEs and trial-specific SAEs and AEs.

In addition to the GCP SAE reporting requirements, for the purposes of this trial, the following events will also be considered SAEs:

- Detention within hospitals using the Mental Health Act.
- Increasing suicidal ideation and/or plans or actual attempts to harm oneself with associated suicidal intent.

The following will be considered AEs:

- Deliberate self-harm which is not life-threatening nor associated with suicidality as judged by the treating clinician.
- A deterioration in mental state is defined as increased anxiety, low mood, aggression, or new evidence of thought disorder and/or perceptual disturbances as judged by the treating clinician.
- Disclosure of a history of physical and/or sexual abuse and/or criminal exploitation.
- Imprisonment.
- Removal from the family home.
- Safeguarding risk to the young person has increased during their participation in the trial to such an extent that the Local Authority (LA) has had to initiate care proceedings.

#### Causality

Causal relationship will be assessed for the SFBT intervention. The PI (or another delegated qualified person from the trial team) will assess each SAE to determine the causal relationship and the CI (or another appropriately qualified member of the TMG) can also provide this assessment where necessary.

#### Expectedness

The CI (or another delegated appropriately qualified individual) will assess each SAE to perform the assessment of expectedness.

Expected events (AE) will be:

- Increased expression of emotion (e.g. crying) during sessions with a therapist.

This event does not need to be reported as an AE.

#### Reporting procedures

The PI will perform the seriousness and causality assessments and return the SAE CRF to Cardiff University within 24 hours of knowledge of the event

The PI will be required to respond to and clarify any queries raised on any reported SAEs and report any additional information as and when it becomes available through to the resolution of the event.

SAEs should be reported throughout the treatment period up to 28 days after the participant receives the intervention.

SAEs will be evaluated by staff at Cardiff University and sent to the CI (or their delegate) for an assessment of expectedness.

Related and unexpected SAEs will be submitted to the REC. These should be sent within 15 days of the CI becoming aware of the event.

#### Urgent safety measures (USMs)

An USM is an action that the Sponsor, CI or PI may carry out in order to protect the participants of a trial against any immediate hazard to their health or safety. Any urgent safety measure relating to this trial must be notified to the REC immediately by telephone, and in any event within 3 days in writing, that such a measure has been taken. USMs reported to Cardiff University will be handled according to Cardiff University processes.

### Frequency and plans for auditing trial conduct {23}

The clinical trial risk assessment has been used to determine the intensity and focus of central and on-site monitoring activity in the trial. Low monitoring levels will be employed and will be fully documented in the trial monitoring plan.

Findings generated from on-site and central monitoring will be shared with the Sponsor, CI, PI and local Research and Development (R&D) department.

### Plans for communicating important protocol amendments to relevant parties (e.g. trial participants, ethical committees) {25}

Changes to the protocol will be communicated with the ethics committee via the Integrated Research Application System (IRAS) amendments process. Participants will be informed of changes to the protocol that will have a significant effect on them via the trial website and email. The trial registry (ISRCTN) and trial sites will be updated with any changes to the protocol.

### Dissemination plans {31a}

A publications plan and policy will be written for the trial and approved by the TMG. Outputs from the trial will include open access peer-reviewed journal articles in international academic journals, at national and international academic conferences and at University public engagement events. All publications and presentations relating to the trial will be authorised by the TMG. A summary of results will be provided to all participants in a format that is suitable for a non-academic audience. Trial results will be published on our website — URL and advice on this will be provided on the PIS. At least one end-of-project dissemination event aimed at CYP and their families, delivery partners, commissioners, and policy makers will be conducted. Various modes of communication will be used to share findings with CYP and their families, these include videos about the study findings, summaries of findings in newsletters/bulletins for participants, infographics or pictorial descriptions of findings. These will be co-produced with the PAG.

## Discussion

This two-arm individually randomised controlled trial will evaluate the effectiveness of SFBT in reducing offending behaviours in CYP presenting at police custody suites in the geographical region served by LSCFT in England. Our process evaluation will assess the acceptability of SFBT in CYP aged 10-17 years, the fidelity of delivery and therefore the overall scalability. This manuscript details the proposed study design; any amendments to the study design, and full justification, will be included in future publications.

## Trial status

Protocol version 1.5 23.03.2023. Recruitment began on 01.02.2023 and due to end on 31.01.2024.

## Abbreviations

CYP: Children and young people
L&D: Liaison and Diversion
SFBT: Solution Focused Brief Therapy
RCT: Randomised controlled trial
SAU: Service as usual
LSCFT: Lancashire and South Cumbria NHS Foundation Trust
SRDM: Self-Report Delinquency Measure
SDQ: Strengths and Difficulties Questionnaire
T-GARM: Gang Affiliation Measure
VCI: Verbal Comprehension Index
PIS: Participant Information Sheets
CF: Consent/Agreement Forms
ONS: Office for National Statistics
SRS: Secure Research Service
PAG: Project Advisory Group
PNC: Police National Computer
WASI-II: Wechsler Abbreviated Scale of Intelligence-II
CRF: Case Report Form
DOB: Date of Birth
MDES: Minimally Detected Effect Size
MoJ: Ministry of Justice
DfE: Department for Education
ADRUK: Administrative Data Research UK
SOP: Standard Operating Procedure
GDPR: General Data Protection Regulation
PID: Participant Identification Number
GLM: Generalised linear models
GLMM: Generalised linear mixed model
SAP: Statistical Analysis Plan
TSC: Trial Steering Committee
CI: Chief Investigator
TMF: Trial Master File
DMC: Data Monitoring Committee
REC: Research Ethics Committee
PI: Principal Investigator
SAE: Serious adverse event
AE: Adverse event
GCP: Good Clinical Practice
LA: Local Authority
USM: Urgent safety measure
R&D: Research and Development
IRAS: Integrated Research Application System
